# Spontaneous Distal Biceps Tendon Rupture as a Rare Finding of Systemic Amyloidosis and Heart Failure with Preserved Ejection Fraction

**DOI:** 10.4274/balkanmedj.galenos.2020.2020.5.67

**Published:** 2020-10-23

**Authors:** Umut Kocabaş, Uğur Önsel Türk

**Affiliations:** 1 Clinic of Cardiology, Başkent University İstanbul Hospital, İstanbul, Turkey; 2 Clinic of Cardiology, KardiyoRitm Cardiac Health Centre, İzmir, Turkey

A 61-year-old man with a history of hospitalization for acute decompensated heart failure and pulmonary edema was admitted to the intensive cardiac care unit with fatigue, shortness of breath, and bilateral leg edema. During his previous hospitalization period, he had carried out a diagnostic coronary angiography for evaluation of the etiology of his heart failure, which revealed patent coronary arteries. Upper extremity musculoskeletal examination showed bunching of the biceps when the patient flexed his arm, which is compatible with rupture of the distal biceps tendon (Figure 1A, Video 1). Twelve-lead electrocardiogram revealed a low QRS voltage and ST-T segment abnormalities (Figure 1B). Echocardiography showed normal ejection fraction with massive ventricular hypertrophy (Figure 1C). Also, echocardiographic tissue Doppler imaging showed restrictive filling patterns with E/A ratio >2.0 and E/e’ ratio >15, which are consistent with grade III diastolic dysfunction (Figure 1D, E). The finding including biceps tendon rupture, QRS hypovoltage, and massive ventricular hypertrophy with grade III diastolic dysfunction were considered highly suggestive of cardiac amyloidosis. In the hematologic diagnostic work-up, monoclonal globulins were detected in both serum and urine protein electrophoresis, which was determined as lambda light chains using immunofixation electrophoresis. The patient was diagnosed as AL amyloidosis with cardiac and musculoskeletal involvement. Written informed consent was obtained from the patient.

Biceps tendon rupture is an infrequent clue for systemic amyloidosis with musculoskeletal involvement (1). In addition, the presence of acute heart failure with massive ventricular hypertrophy and normal ejection fraction, grade III diastolic dysfunction, QRS hypovoltage, and spontaneous biceps tendon rupture should raise the clinical suspicion of systemic amyloidosis.

## Figures and Tables

**Figure 1:**
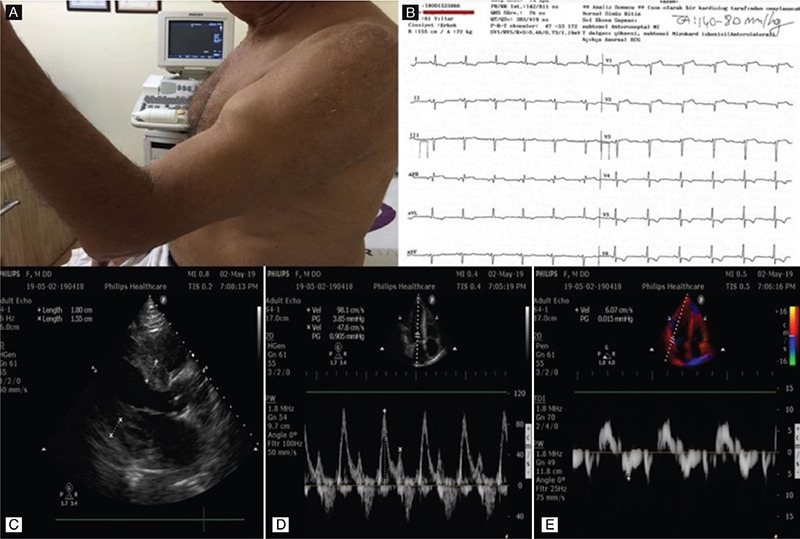
Clinical findings of the patient. Upper extremity musculoskeletal examination was compatible with biceps tendon rupture (A), 12-lead electrocardiogram revealed low QRS voltage and ST-T segment abnormalities (B), echocardiographic imaging revealed massive hypertrophy (C), and grade III diastolic dysfunction (D,E).
